# Effect of Eugenol against *Streptococcus agalactiae* and Synergistic Interaction with Biologically Produced Silver Nanoparticles

**DOI:** 10.1155/2015/861497

**Published:** 2015-04-07

**Authors:** Renata Perugini Biasi-Garbin, Eliane Saori Otaguiri, Alexandre Tadachi Morey, Mayara Fernandes da Silva, Ana Elisa Belotto Morguette, César Armando Contreras Lancheros, Danielle Kian, Márcia Regina Eches Perugini, Gerson Nakazato, Nelson Durán, Celso Vataru Nakamura, Lucy Megumi Yamauchi, Sueli Fumie Yamada-Ogatta

**Affiliations:** ^1^Departamento de Microbiologia, Centro de Ciências Biológicas, Universidade Estadual de Londrina, Rodovia Celso Garcia Cid, PR 445, Km 380, 86057-970 Londrina, PR, Brazil; ^2^Programa Nacional de Pós Doutorado (PNPD/CAPES), Departamento de Microbiologia, Centro de Ciências Biológicas, Universidade Estadual de Londrina, Rodovia Celso Garcia Cid, PR 445, Km 380, 86057-970 Londrina, PR, Brazil; ^3^Departamento de Patologia, Análises Clínicas e Toxicológicas, Laboratório de Microbiologia Clínica, Centro de Ciências da Saúde, Universidade Estadual de Londrina, Avenida Robert Koch 60, Vila Operária, 86038-350 Londrina, PR, Brazil; ^4^Instituto de Química, Universidade Estadual de Campinas, Cidade Universitária Zeferino Vaz, s/n Barão Geraldo, Caixa Postal 6154, 13085-970 Campinas, SP, Brazil; ^5^Departamento de Ciências Básicas da Saúde, Centro de Ciências da Saúde, Universidade Estadual de Maringá, Avenida Colombo 5790, Jardim Universitário, 87020-900 Maringá, PR, Brazil

## Abstract

*Streptococcus agalactiae* (group B streptococci (GBS)) is an important infections agent in newborns associated with maternal vaginal colonization. Intrapartum antibiotic prophylaxis in GBS-colonized pregnant women has led to a significant reduction in the incidence of early neonatal infection in various geographic regions. However, this strategy may lead to resistance selecting among GBS, indicating the need for new alternatives to prevent bacterial transmission and even to treat GBS infections. This study reported for the first time the effect of eugenol on GBS isolated from colonized women, alone and in combination with silver nanoparticles produced by *Fusarium oxysporum* (AgNPbio). Eugenol showed a bactericidal effect against planktonic cells of all GBS strains, and this effect appeared to be time-dependent as judged by the time-kill curves and viability analysis. Combination of eugenol with AgNPbio resulted in a strong synergistic activity, significantly reducing the minimum inhibitory concentration values of both compounds. Scanning and transmission electron microscopy revealed fragmented cells and changes in bacterial morphology after incubation with eugenol. In addition, eugenol inhibited the viability of sessile cells during biofilm formation and in mature biofilms. These results indicate the potential of eugenol as an alternative for controlling GBS infections.

## 1. Introduction

*Streptococcus agalactiae* (Group B* Streptococcus* (GBS)) is an important cause of invasive diseases, mainly in newborns, pregnant women, and elderly individuals [1, 2]. Neonatal early onset diseases, which are characterized by sepsis, pneumonia, or meningitis, are strongly associated with maternal vaginal colonization and may occur vertically by aspiration of infected amniotic fluid or during passage through the birth canal [3]. It is estimated that 10–37% of pregnant women are colonized with GBS, and in the absence of any intervention, 30–70% of newborns become colonized, of which about 1–3% develop invasive diseases with high mortality rates [3, 4].

The active prevention strategy for GBS neonatal infections based on bacterial screening and intrapartum antibiotic prophylaxis (IAP) has led to a significant reduction in the incidence of early neonatal infection in various regions of the world. Current IAP consists of intravenous administration of beta-lactams (penicillin or ampicillin) to GBS-colonized pregnant woman for 4 h before delivery. Erythromycin, clindamycin, and vancomycin are the agents recommended in cases of colonization during pregnancy with beta-lactam-resistant GBS or beta-lactam allergy [4].

In general, GBS isolates have been shown to be sensitive to beta-lactams [5–7]. However, GBS isolates with reduced susceptibility and even resistance to beta-lactam agents have been reported in recent years [8, 9]. Moreover, resistance to erythromycin and clindamycin among GBS isolates has emerged in different regions of the world, with rates ranging from 14.5% to 70% and 8.2% to 70% for erythromycin and clindamycin, respectively [10–12].

Besides the emergence of GBS resistant to current antibiotics, the development of vaccines against GBS is still in progress, indicating that new safe and effective alternatives should be developed to prevent the transmission of this bacterium. Several authors have described the antibacterial activity of natural products against GBS [13–15], indicating their potential as alternatives for IAP, reducing the risk of development of resistance to commercially available antibiotics.

Eugenol, a major constituent of essential oils extracted from various plants, has been widely studied due to its medicinal properties, such as the following: antibacterial [16–18], antifungal [19], antiviral [20], antileishmanial [21], antioxidant [22], anticarcinogenic [23], and analgesic and anti-inflammatory [24]. Similarly, silver nanoparticles have been extensively investigated because of their antimicrobial properties alone [25, 26] or in combination with other compounds [27–29].

In this study, we evaluated the antibacterial effect of the phenylpropanoid eugenol on planktonic cells and biofilm of GBS isolated from colonized women, including those exhibiting resistance to erythromycin and clindamycin and its synergistic interaction with silver nanoparticles produced by* Fusarium oxysporum* (AgNPbio).

## 2. Material and Methods

### 2.1. Bacteria and Growth Conditions

Six nonduplicates strains of* Streptococcus agalactiae* (GBS) recovered from vaginal-rectal swabs of colonized women were taken from the bacterial collection of the Laboratory of Clinical Microbiology of University Hospital of Londrina, Paraná, Brazil. Bacterial strains were included according to phenotypic and genotypic characteristics [11] (Table 1). Bacteria were kept at −20°C in tryptic soy broth (TSB, Oxoid) containing 20% glycerol and 5% sheep blood. The reference strain* S. agalactiae* ATCC 13813 (kindly donated by FIOCRUZ, Rio de Janeiro, Brazil) was also included in this study. Bacteria were grown in TSB, pH 6.5, at 37°C for 24 h before the assays. The study protocol was approved by the Ethics Committee of the Universidade Estadual de Londrina (document 186/09-CEP/UEL). Written informed consent was obtained from the patients for the publication of this report and any accompanying images.

### 2.2. Eugenol and Biologically Synthesized Silver Nanoparticles

A stock solution of 10% eugenol (SSWhite, Brazil) was prepared in TSB, pH 6.5, containing 10% (v/v) dimethyl sulfoxide (DMSO; Sigma Chemical Co., USA). The DMSO final concentration in the assays did not exceed 1.0%. Biological silver nanoparticles (AgNPbio) were obtained after AgNO_3_ reduction by* F. oxysporum* as previously described [30]. Briefly,* F. oxysporum *was cultured in broth medium containing 0.5% yeast extract at 28°C for 6 days. The fungal biomass was filtered, added (approximately 10 g) to 100 mL of distilled water, and incubated at 28°C for 72 h. A fungal-free solution was obtained by filtration and mixed with 1.0 mM AgNO_3_ and the mixture was kept at 28°C for 28 h. AgNPbio were purified and characterized by scanning electron microscopy and energy-dispersive spectroscopy.

### 2.3. Antibacterial Activity of Eugenol against Planktonic Cells

The minimal inhibitory concentration (MIC) of eugenol was determined by broth microdilution method according to the Clinical and Laboratory Standards Institute [31], with modifications. Twofold serial dilutions of eugenol (1–0.015%) in TSB were prepared in sterile polystyrene 96-well plates (Techno Plastic Products, Switzerland). Wells contained medium alone or medium plus 1% DMSO and untreated planktonic cells, and medium alone served as growth and sterility controls. After 24 h incubation, optical density was measured at 600 nm with a microtiter plate reader (Synergy HT, Biotek) and MIC was determined at total inhibition of growth after 24 h incubation compared to untreated planktonic cells [32]. MICs for erythromycin and clindamycin were also determined as described above, and* Streptococcus pneumoniae* ATCC 49619 was used as the quality control. To determine the minimal bactericidal concentration (MBC), the content from the wells (10 *μ*L) showing no growth was transferred to plates with tryptic soy agar (TSA, Oxoid) containing 5% sheep blood and incubated at 37°C for 24 h. MBC was defined as 100% decrease in colony forming units (CFU) compared to untreated bacteria. All assays were carried out in triplicate on two different occasions.

### 2.4. Time-Kill Curves

For time-kill curve analysis, planktonic cells of GBS 89, GBS 121, and* S. agalactiae* ATCC 13813 (1–5 × 10^5^ CFU/mL) were incubated in TSB containing MIC levels of eugenol. At determined time points (0, 1, 2, 4, 6, 8, 10, and 24 h), aliquots were aseptically transferred to TSA plus 5% sheep blood plates and the CFU counts were determined after incubation at 37°C for 24 h. The bacterial viability of eugenol-treated GBS was also evaluated, using LIVE/DEAD* Bac*Light staining kit (Molecular Probes, Invitrogen) according to the manufactures' recommendations. This assay is based on the detection of two nucleic acid fluorescent stains. Green-fluorescent SYTO 9 labels live and dead bacteria, whereas the red fluorescent propidium iodide selectively labels bacteria with permeable (damaged) membranes. Bacterial cells were placed on a glass coverslip and analyzed by fluorescence microscopy (LEICA DM2000). All assays were carried out in triplicate on three different occasions.

### 2.5. Antibacterial Activity of Eugenol against Biofilms

Biofilms of GBS strains and* S. agalactiae* ATCC 13813 were formed on polystyrene surface of flat-bottomed 96-well microtiter plates (Techno Plastic Products, Switzerland) in a static model at 37°C according to Borges et al. [33]. Briefly, bacterial cells were grown in TSB at 37°C for 18 h, harvested by centrifugation, and washed with sterile 0.15 M phosphate-buffered saline (PBS), pH 7.2, and the cell density was adjusted to 1.0 × 10^8^ CFU/mL in the same medium. Twofold serial dilutions of eugenol (2–0.03%) were prepared in TSB. For analysis of eugenol effect during biofilm formation, a 20 *μ*L aliquot of each cell suspension was transferred to each well containing 180 *μ*L of TSB with different concentrations of eugenol, and the plates were incubated for 24 h. For analysis of eugenol effect on mature biofilm, 20 *μ*L of each cell suspension was added to each well containing 180 *μ*L of TSB. After 24 h of biofilm formation, the medium was aspirated off and each well was washed with sterile PBS. A 200 *μ*L aliquot of TSB containing different concentrations of eugenol was added and the plates were incubated for another 24 h. Controls included eugenol-free wells and biofilm-free wells. Sessile (biofilm) minimum inhibitory concentrations were determined at 50 and 100% inhibition (SMIC) compared to eugenol-free control wells using the 2,3-bis(2-methoxy-4-nitro-5-sulfo-phenyl)-5-[(phenylamino)carbonyl]-2*H*-tetrazolium hydroxide (XTT)-reduction assay [34]. A 200 *μ*L aliquot of XTT-menadione [0.5 mg/mL XTT, 1 mM menadione (Sigma Chemical Co., USA)] was added to each well, and the plates were incubated in the dark at 37°C for 90 min, after which the optical density was measured at 490 nm with a microtiter plate reader (Synergy HT, Biotek). Experiments were carried out in quintuplicate on two different occasions.

### 2.6. Electron Microscopy Analysis

Planktonic cells treated with MIC levels of eugenol for 5 h were fixed for 2 h at room temperature with 2.5% glutaraldehyde in 0.1 M sodium cacodylate buffer, pH 7.2. For scanning electron microscopy (SEM), postfixed cells were dehydrated with a series of ethanol washes (15, 30, 50, 70, 80, 90, 95, and 100%), critical-point dried in CO_2_, coated with gold, and examined with a SHIMADZU SS-550 scanning electron microscope. For transmission electron microscopy (TEM), postfixation was carried out in 1% osmium tetroxide in cacodylate buffer containing 0.8% potassium ferrocyanide and 5 mM CaCl_2 _at room temperature for 1 h. The cells were dehydrated in acetone and embedded in Epon resin. Ultrathin sections were stained with uranyl acetate and lead citrate and examined with a Zeiss EM900 electron microscope.

### 2.7. Analysis of the Combinations of Eugenol and AgNPbio

The effect of eugenol combined with AgNPbio on planktonic cells of GBS was assessed by checkerboard method in 96-well microtiter plates, as previously described [35]. Twofold serial dilutions of the compounds were prepared in TSB, and the final concentrations of eugenol and AgNPbio ranged from 1 to 0.015% and 500 to 0.49 *μ*M, respectively. Bacteria (1.0 × 10^5^ cells) were grown with eugenol or AgNPbio individually and in combinations at 37°C for 24 h. The interactions of the two compounds were analyzed by the fractional inhibitory concentration index (FICI), which was defined as the sum of FIC_eugenol_ and FIC_AgNPbio_. FIC of the material is the concentration that kills when being used in combination with another divided by the concentration that has same effect when used individually [35].

### 2.8. Statistical Analysis

The results were evaluated by one-way ANOVA using the GRAPHPAD PRISM version 5.0 (GRAPHPAD Software, San Diego, CA). *P* < 0.05 was considered significant.

## 3. Results

### 3.1. Antibacterial Activity of Eugenol against Planktonic Cells

**Figure 1:**
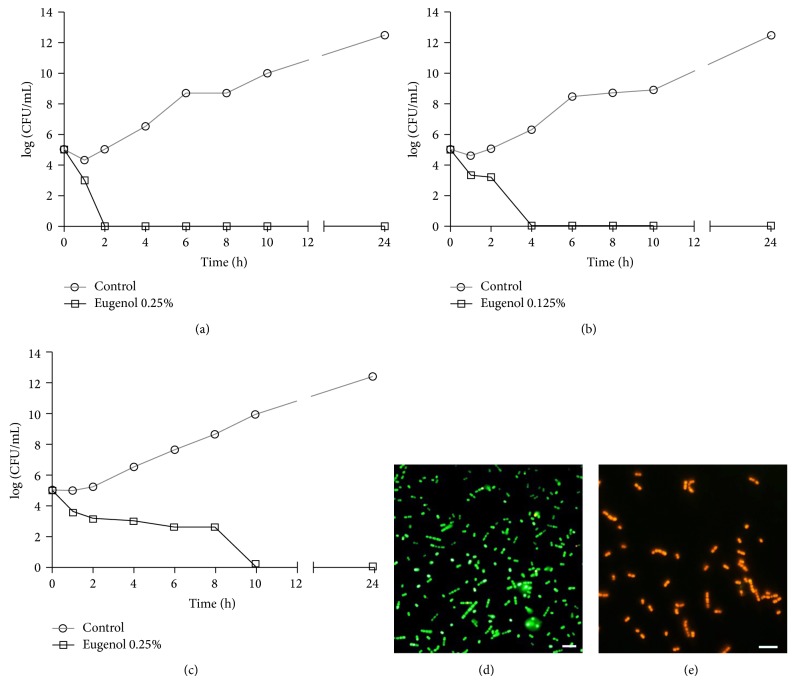
Effect of eugenol on growth (a–c) and viability (d-e) of* Streptococcus agalactiae*. Time-kill curves of GBS 89 (a), GBS 121 (b), and* S. agalactiae* ATCC 13813 (c) strains: bacteria were incubated with eugenol at MIC for 24 h at 37°C and the CFU counts were determined at specified time points. Viability of the cells was determined with live-dead staining and GBSs with intact membranes were green-fluorescent (d), whereas eugenol-treated GBSs (at MIC) with damaged membranes were red-fluorescent (e). Representative images are shown. Bar = 5 *μ*m.

Eugenol inhibited the planktonic growth of all GBS strains, including those resistant to erythromycin and/or clindamycin. MIC and MBC values were exactly at the same concentrations for all GBS strains analyzed in this study. The mean MIC/MBC value was 0.23 ± 0.13% ranging from 0.125 to 0.5% (Table 1). To evaluate the killing kinetics of eugenol against GBS, survival of planktonic cells from GBS 89, GBS 121, and reference strain was assessed during 24 h in the presence of the phenylpropanoid at MIC. As shown in Figure 1, a time-dependent bactericidal effect was observed for all strains. The presence of eugenol at MIC reduced the planktonic cell population approximately by 1 to 2 log⁡_10_⁡ CFU/mL (*P* < 0.05) after one hour of incubation. No colony forming units (Figures 1(a), 1(b), and 1(c)) or cellular viability (Figures 1(d) and 1(e)) was detected after 2, 4, and 10 h of incubation in the presence of eugenol for GBS 89, GBS 121, or reference strain. At the specified time, all eugenol-treated GBSs were red-fluorescent, reflecting dead bacteria with damaged membranes, in contrast to green-fluorescent untreated cells.

The combination of eugenol with AgNPbio significantly reduced (*P* < 0.05) the MIC value of both compounds, and the calculated FICI indicated a synergistic effect between them against all GBS strains (Table 2). AgNPbio alone at 125 *μ*M inhibited the growth of all GBS strains. When the two compounds were combined, the MIC values of eugenol and AgNPbio decreased 4- to 8-fold and 4- to 256-fold, respectively.

### 3.2. Morphological and Ultrastructural Alterations in Eugenol-Treated Planktonic Cells

**Figure 2:**
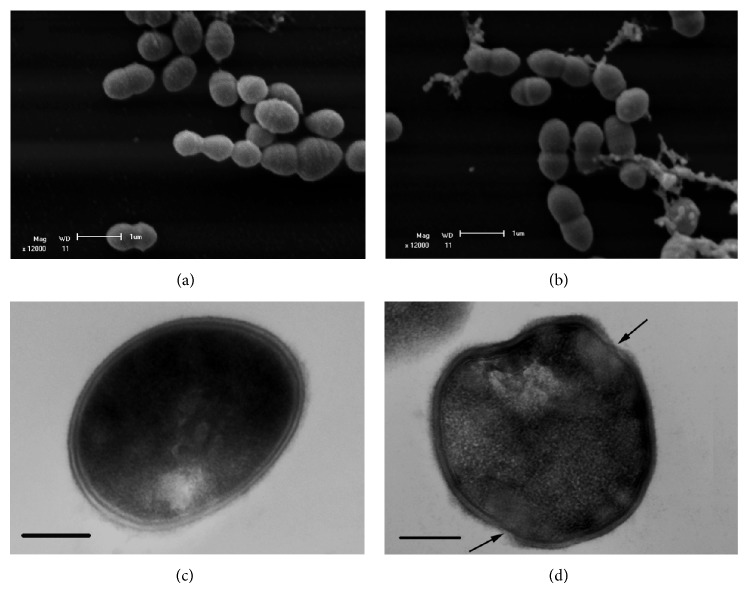
Scanning electron microscopy (a and b) and transmission electron microscopy images (c and d) of the effect of eugenol on* Streptococcus agalactiae* ATCC 13813. Untreated cells (a and c) and treated cells with eugenol 0.25% for 5 h (b and d). Bar: (a and b) = 1 *μ*m, (c and d) = 200 nm.

SEM images showed untreated control cells with typical morphology arranged in an organized manner (Figure 2(a)). In contrast, the incubation of GBS planktonic cells with an inhibitory concentration of eugenol for 5 h led to cell lysis resulting in leakage of cytoplasmic contents (Figure 2(b)). Likewise, TEM imaging of control cells showed an intact cell wall with regular electron density (Figure 2(c)) and treated cells displaying various alterations such as changes in bacterium morphology; disruption of cell wall (arrow), corroborating the SEM results; and decrease in electron density (Figure 2(d)).

### 3.3. Antibacterial Activity of Eugenol in Biofilms

Eugenol inhibited biofilm formation of GBS, and no metabolic activity was detected at concentrations ranging from 0.06 to 0.5%, where these values were considered, SMIC_100_ (Table 1). In addition, eugenol decreased significantly the viability of mature (24 h) biofilm at concentrations ranging from 0.03 to 2%, with SMIC_50_ ranging from 0.02 to 0.25% (Table 1). However, except for the reference strain, no total inhibition was observed at the highest concentration tested in this study. At 2% eugenol, reduction in metabolic activity ranged from 60 to 90% for the other GBS strains, where GBS 121 biofilm was the least susceptible.

## 4. Discussion

Eugenol is a remarkably versatile molecule, which can be incorporated as a functional compound in various products applied not only in the pharmaceutical industry but also in the agricultural, food, and cosmetic industries as well [36].

In this study, the bactericidal activity of eugenol against human isolates of* S. agalactiae*, including those exhibiting different mechanisms of resistance to erythromycin and/or clindamycin, is reported for the first time. The antibacterial activity of eugenol against planktonic cells of different species of the genus* Streptococcus* has been previously reported [35, 37–39]. Baskaran et al. [37] reported the bactericidal activity of eugenol against planktonic cells of five agents of bovine mastitis, including* S. agalactiae*, whose MIC and MBC were 0.4 and 0.8%, respectively.

The bactericidal effect of eugenol on* S. agalactiae* seems to be dependent on changes in the cell envelope, as judged by alterations in the morphology and ultrastructure observed in treated cells. In fact, other authors reported that eugenol induces cell lysis through protein and lipid leakage, leading to extrusion of cytoplasmic content in various Gram-negative and Gram-positive bacteria, including* Streptococcus pyogenes* [17, 35, 38]. In addition, eugenol is capable of inhibiting the membrane-bound ATPase activity of* Escherichia coli* and* Listeria monocytogenes* [40].

In this study, silver nanoparticles synthesized by an ecofriendly method using the filamentous fungus* F. oxysporum* showed inhibitory activity against planktonic cells of all GBS strains. Moreover, the synergistic antibacterial interaction of AgNPbio with eugenol against GBS is reported for the first time. Metallic nanoparticles have been widely studied because of their broad spectrum antimicrobial effect, even at low concentrations [25–28]. In addition, the combination of these nanoparticles with several compounds has shown potent antimicrobial activity in different microbial species, including those displaying resistance to conventional antibiotics [26–29]. Accordingly, additive or synergistic effect of essential oil component cinnamaldehyde with chemically synthesized silver nanoparticles against Gram-positive and Gram-negative bacteria has been reported elsewhere [29].

Current antibacterial agents have limited efficacy on biofilms. Sessile (adhered) bacteria in these communities have different physiological characteristics compared to free-floating planktonic cells. Clinically, these features can result in protection against the host immune system and less susceptibility to antimicrobial agents, contributing to persistent infections and difficult treatment [41]. Biofilms are also established during host colonization, enabling the bacteria to withstand removal by mechanical processes [42]. Except for* S. agalactiae*, the antibiofilm activity of eugenol for various bacterial species has been reported elsewhere [35, 43]. In this study, eugenol also exhibited an antibacterial activity against biofilms of* S. agalactiae*, showing the ability to inhibit its formation as well as the viability of mature biofilm, under* in vitro* conditions. Similarly, Yadav et al. [35] reported the inhibitory effect of eugenol against biofilms of* S. pneumoniae*. Furthermore, Adil et al. [44] described that* Streptococcus mutans* incubated in the presence of subinhibitory concentrations of eugenol showed decreased expression of genes related to biofilm formation.

A limitation of this study, which may reduce the generalization of the results, is the number of GBS strains. Despite this limitation, the results presented here showed the antibacterial activity of eugenol against* S. agalactiae*, alone or in combination with AgNPbio, opening a promising strategy for application in IAP for women colonized with this bacterium. In this sense, a variety of evidence supports the application of eugenol in human healthcare. The addition of eugenol and other essential oils in animal feed reduced the* Clostridium perfringens* load in the gut of broiler chickens. Although this bacterium can be a harmless intestinal inhabitant of chickens, it is the leading agent of necrotic enteritis in these hosts [45]. Topical application of eugenol on teeth reduced the incidence and severity of carious lesions caused by* S. mutans* in rats [39]. Preliminary* in vivo* studies have shown the safety and efficacy of topical use of eugenol in combination with thymol in the treatment of bacterial vaginosis and vaginal candidiasis [46]. The incorporation of eugenol in polymeric material has been shown to reduce the biofilm formation on surfaces [43].

## 5. Conclusion

The results obtained in this study demonstrated the bactericidal activity of eugenol and its synergistic effect with AgNPbio against planktonic cells of* S. agalactiae*. Furthermore, this compound inhibited biofilm formation and viability of mature biofilm formed on polystyrene.

## Figures and Tables

**Table 1:** Phenotypic and genotypic characteristics and antibacterial susceptibility profile of eugenol for planktonic and sessile cells of *Streptococcus agalactiae*.

GBS strains	MLVA genotypes^a^	Capsular types^a^	E (*µ*g/mL)	DA (*µ*g/mL)	Antimicrobial resistance genes^a^	Eugenol (%)		
			MIC^b^			MIC^b^/MBC^c^	SMIC_100_^d^	SMIC_50_^e^
50	8	Ia	0.25	0.25	—	0.5	0.25	0.09
72	5	III	0.125	0.25	—	0.125	0.25	0.06
80	6	V	0.25	0.125	—	0.25	0.125	0.05
89	13	Ia	0.25	0.125	—	0.25	0.25	0.09
115	7	V	>1024	1024	*ermB *	0.125	0.5	0.02
121	8	Ia	16	0.125	*mefA/E *	0.125	0.5	0.25
ATCC 13813	—	—	0.125	0.125	—	0.25	0.06	0.04

^a^The genetic diversity, the capsular type, and the resistance genes were previously determined by Otaguiri et al. [11]. ^b^Minimum inhibitory concentration (MIC) of the compound which resulted in total inhibition of visible planktonic cell growth defined according to CLSI (2012) [31] guidelines by broth microdilution assays. ^c^Minimum bactericidal concentration (MBC) of the eugenol. ^d^Sessile MIC (SMIC) of the eugenol which resulted in total reduction in metabolic activity of sessile cells during biofilm formation, using the XTT-reduction assay, after 24 h. ^e^SMIC of the eugenol which resulted in 50% of reduction in metabolic activity of sessile cells from mature biofilm (24 h), using the XTT-reduction assay. MLVA: multiple *locus* variable number of tandem repeat analysis; E: erythromycin; DA: clindamycin.

**Table 2:** *In vitro* synergistic effect of eugenol and biological silver nanoparticles on *Streptococcus agalactiae *growth.

GBS strains	MIC^a^			FICI^d^	Effect
	Eugenol^b^ (%)	AgNPbio^c^ (*µ*M)	Eugenol/AgNPbio		
50	0.5	125	0.06/15.62	0.25	Synergism
72	0.125	125	0.03/31.25	0.5	Synergism
80	0.25	125	0.06/0.49	0.25	Synergism
89	0.25	125	0.03/7.8	0.19	Synergism
115	0.25	125	0.03/31.25	0.38	Synergism
121	0.125	125	0.03/3.9	0.281	Synergism
ATCC 13813	0.25	125	0.03/15.62	0.25	Synergism

^a^MIC: minimum inhibitory concentration. ^b^MIC of eugenol used alone. ^c^MIC of silver nanoparticle used alone. ^d^FICI: fractional inhibitory concentrations index were calculated according to Yadav et al. [35] and classified as follows: synergistic if FIC ≤0.5, additive if FIC >0.5 and ≤1.0, indifferent if FIC >1.0 and ≤2.0, and antagonistic if FIC >2.0.
